# Blood sLOX-1 Is Beneficial for Determining Severity of Neonatal Hypoxic–Ischemic Encephalopathy: A Nationwide Prospective Cohort Study

**DOI:** 10.3390/medsci14030391

**Published:** 2026-07-14

**Authors:** Takuya Oshima, Yoshinori Aoki, Tomohisa Akamatsu, Yutaka Matsuyama, Naoto Takahashi, Toshimasa Obonai, Masaki Shimizu, Kaoru Okazaki, Jun Shibasaki, Osuke Iwata, Takafumi Sakakibara, Reiko Kushima, Kenichi Masumoto, Masahiro Kinoshita, Daigo Kajikawa, Yumi Kono, Yasuki Maeno, Ken Nagaya, Kentaro Shirai, Atsushi Naito, Akira Oka, Masayuki Itoh

**Affiliations:** 1 Department of Biochemistry and Cellular Biology (Mental Retardation and Birth Defect Research), National Center of Neurology and Psychiatry, Tokyo 187-8502, Japan; 2 Department of Pediatrics, Faculty of Medicine, The University of Tokyo, Tokyo 113-8655, Japan; 3 Department of Pediatrics, National Center for Global Health and Medicine, Tokyo 162-8655, Japan; 4 Department of Biostatistics, School of Public Health, Graduate School of Medicine, The University of Tokyo, Tokyo 113-8655, Japan; 5 Department of Pediatrics, Tokyo Metropolitan Tama-Hokubu Medical Center, Tokyo 189-8511, Japan; 6 Department of Neonatology, Saitama Prefectural Children’s Medical Center, Saitama 330-8777, Japan; 7 Department of Neonatology, Tokyo Metropolitan Children’s Medical Center, Tokyo 130-8575, Japan; 8 Department of Neonatology, Kanagawa Children’s Medical Center, Kanagawa 232-8555, Japan; 9 Department of Pediatrics and Neonatology, Nagoya City University, Nagoya 467-8601, Japan; 10 Department of Pediatrics, Nara Medical University, Kashihara 634-8521, Japan; 11 Department of Neonatology, Tokyo Metropolitan Bokutoh Hospital, Tokyo 130-8575, Japan; 12 Department of Neonatology, Faculty of Medicine, Toho University, Tokyo 143-8540, Japan; 13 Department of Pediatrics and Child Health, Kurume University School of Medicine, Fukuoka 830-0011, Japan; 14 Department of Neonatology, Ibaraki Children’s Hospital, Ibaraki 311-4145, Japan; 15 Department of Pediatrics, Jichi Medical University, Shimotsuke 329-0498, Japan; 16 Division of Neonatology, St. Mary’s Hospital, Kurume 830-8543, Japan; 17 Department of Pediatrics, Asahikawa Medical University, Asahikawa 078-8510, Japan; 18 Department of Pediatrics, Tsuchiura Kyodo General Hospital, Tsuchiura 300-0028, Japan; 19 Department of Neonatology, Yamanashi Prefectural Central Hospital, Yamanashi 400-8506, Japan; 20 Saitama Prefectural Children’s Medical Center, Saitama 330-8777, Japan

**Keywords:** neonatal hypoxic–ischemic encephalopathy (HIE), severity, biomarker, soluble form of lectin-like oxidized low-density lipoprotein receptor-1 (sLOX-1)

## Abstract

**Background/Objectives:** Neonatal hypoxic–ischemic encephalopathy (HIE) is a major cause of neurodevelopmental diseases. In a previous retrospective study, we revealed that the levels of the soluble form of lectin-like oxidized low-density lipoprotein receptor-1 (sLOX-1) are linked to HIE severity. We conducted a prospective investigation to evaluate the association between sLOX-1 levels and HIE severity using the Sarnat staging system, as well as a multicenter study to assess neurodevelopmental sequelae in the short term. **Methods**: A total of 193 infants were enrolled in the study between April 2018 and March 2021. We divided the infants into groups as follows: infants without HIE were assigned to a normal control group, and those with HIE to mild, moderate, and severe HIE groups. We measured 191 samples, excluding three participants. The distribution of 191 participants was as follows: 88 normal samples of 50 umbilical cord (UC) arteries and 38 venous blood samples (control; CTL), as well as 103 HIE samples of 34 mild, 55 moderate, and 14 severe HIE samples. All the plasma samples were collected until 6 h after birth. **Results:** The mean sLOX-1 value in the CTL group was 613 pg/mL (IQR: 474–925 pg/mL) and that in the UC group was 497 pg/mL (IQR: 337–693 pg/mL), with no significant difference (*p* = 0.136). The mean sLOX-1 value in the mild HIE group was 1030 pg/mL (IQR: 658–1547 pg/mL), 1026 pg/mL (IQR: 710–2051 pg/mL) in the moderate HIE group, and 1444 pg/mL (IQR: 766–3927 pg/mL) in the severe HIE group. There were significant differences between the CTL and HIE groups (*p* = 0.006) and between the CTL and mild HIE groups (*p* < 0.05), but not between the mild HIE and the moderate and severe HIE groups (*p* = 0.088). The other blood markers exhibited no correlation with Sarnat severity. Interestingly, blood sLOX-1 levels increased with Sarnat severity. **Conclusions:** The results of this study indicated that sLOX-1 level was correlated with Sarnat severity. Moreover, early assessment of sLOX-1 level may be useful for deciding whether to induce therapeutic hypothermia and/or other treatments, and sLOX-1 may serve as a short-term outcome biomarker of HIE.

## 1. Introduction

Neonatal hypoxic–ischemic encephalopathy (HIE), which occurs in approximately 1.5 to 2.96 cases per 1000 births each year in developed countries [1,2,3], is a perinatal brain injury that is often associated with death or severe neurodevelopmental impairments in survivors. This pathology leads to major brain damage via acute respiratory and circulatory failure, which occurs following asphyxia in the fetal and/or neonatal period. Although mild and transient brain hypoxia and ischemia are often reversible, severe and prolonged damage can be irreversible and lead to serious brain damage that can progress to cerebral palsy, epilepsy, and various psychomotor developmental problems [1].

Therapeutic hypothermia is currently the only effective treatment option for this condition; however, it only partially improves the mortality rate and neurodevelopmental impairments among infants with moderate and severe HIE. In addition, therapeutic hypothermia needs to be initiated within 6 h after birth. An early decision regarding the severity of the condition is usually made on the basis of the widely used Sarnat classification or its modification [4]. However, neurological assessment using these scales requires specialized knowledge, training, and skills, thereby limiting the pool of examiners to skilled neonatologists and neurologists. On the other hand, studies have indicated that the neuron-specific enolase, S100B, tau, ubiquitin carboxy-terminal hydrolase L1 protein, and brain-derived neurotrophic factor could serve as biomarkers for diagnosing and determining the severity of HIE [5,6]. However, these biomarkers are not sufficient for clinical use in acute-phase assessment and in determining short- or long- term outcomes.

We previously described the soluble form of lectin-like oxidized low-density lipoprotein receptor-1 (sLOX-1) as a potential biomarker [7]. LOX-1 is a scavenger receptor of oxidized low-density lipoprotein (oxLDL) whose expression is induced by oxidant species, inflammatory cytokines, and ischemia–reperfusion in thrombocytes, endothelial cells, neurons, and macrophages [8,9]. Furthermore, sLOX-1 is expressed in the blood and has been used as a predictive factor for acute coronary syndrome, stroke and preeclampsia [10,11,12,13,14,15]. In an HIE model, we revealed that the expression of LOX-1 was increased in microglial cells and the LOX-1-neutralizing antibody was effective for acute-phase treatment [16,17]. Here, we validated whether blood sLOX-1 levels are supportive for assessing HIE severity.

## 2. Materials and Methods

This multicenter prospective observational study, conducted to evaluate the association between sLOX-1 and HIE severity, was approved by the Ethics Committees of the National Center of Neurology and Psychiatry (A2017-113) and the 23 consortium institutions and hospitals. We obtained consent for participation from the parents of all enrolled infants. A total of 193 infants were enrolled in the study between April 2018 and March 2021. The study design was developed as a large prospective cohort study, with the methodology based on that of a previous study [7]. Specifically, the inclusion criteria for the HIE and control groups included a gestational age ≥ 36 weeks and a birth weight ≥ 1800 g. The inclusion criteria for infants with HIE were based on those used for the randomized controlled trial on therapeutic hypothermia in HIE infants conducted by the National Institute of Child Health and Human Development (NICHD); they included a pH ≤ 7.0 or a base deficit ≥ 16 mmol/L in any blood sample obtained within 60 min after birth, as well as a 10 min APGAR score ≤ 5 or resuscitation with assisted ventilation for ≥ 10 min after birth. Infants meeting these criteria were classified as having HIE. Infants in the control group were those who met the inclusion criteria but did not have HIE. The exclusion criteria included chromosomal abnormalities, congenital infections, congenital heart disease, major malformations, and incomplete clinical information.

We classified the infants with HIE into mild, moderate, and severe HIE groups according to the modified Sarnat staging system. We examined neurologic conditions in the following 6 categories: consciousness, spontaneous activity, posture, tone, primitive reflexes (such as sucking and the Moro reflex), and the autonomic nervous system (including pupillary reactions and heart rate). Infants with abnormalities in 3 or more categories were classified as having moderate or severe HIE, and infants with abnormalities in 2 or fewer categories were classified as having mild HIE. Moderate and severe HIE were distinguished by the number of moderate or severe HIE signs, and if the number of signs was equal, the distinction was made on the basis of the infant’s level of consciousness. Therapeutic hypothermia was performed for infants with moderate and severe HIE without severe complications, such as disseminated intravascular coagulation or severe pulmonary hypertension. Neurosensory impairments were defined as feeding disorders (necessitating tube feeding at discharge), hearing disorders, seizures, and severe hypotonia. Intact survival was defined as the absence of these abnormalities at discharge. We performed automated auditory brainstem response (ABR) testing (NatusALGO 3i; Atom Medical, Tokyo, Japan) at 35 dB nHL in all infants at discharge. Infants exhibiting poor responses underwent diagnostic ABR testing (Neuropack S1 MEB 9402; Nihon Kohden, Tokyo, Japan). An infant demonstrating prolonged latencies or indistinct waves at 80 dB SPL on ABR examination was classified as having a hearing disorder.

Plasma samples were obtained at regular intervals until 6 h after birth as available. Blood samples of 0.5 mL were collected from a vein or an artery of neonates in the Neonatal Intensive Care Unit (NICU) and from an umbilical artery of infants in the obstetrics clinic; each sample was collected in an EDTA-2K (ethylenediaminetetraacetic acid dipotassium salt dihydrate) tube and immediately centrifuged for 15 min at 3000 rpm at 4 °C. The supernatant was preserved as a plasma sample at −80 °C until measurements were performed. The sLOX-1 levels were measured via a sandwich enzyme-linked immunosorbent assay (ELISA) using 2 types of monoclonal antibodies against the extracellular domain of LOX-1.

In addition, we collected maternal and neonatal clinical and biological data at birth and discharge, including neurological symptoms of paralysis, abnormal muscle tone, seizure, and death.

Statistical analyses were performed using SPSS Statistics version 24.0 (IBM, Armonk, New York, NY, USA) and SAS version 9.4 (Statistical Analysis System, SAS Institute, Singapore). Statistical analyses were performed on maternal clinical characteristics, clinical information of participants at the time of admission, and items related to participants’ admission and short-term outcomes (Table 1, Table 2 and Table 3, respectively). Categorical variables were compared using the Mantel-trend test, chi-square test, and Fisher test, and continuous variables were compared through analysis of variance (contrasts). Statistical analyses of sLOX-1 values were conducted using log-transformed data to reduce the influence of outliers (Table 4). We performed a multiple regression analysis to examine the differences between groups, and expressed the intergroup differences in interest as contrast coefficients, which were then tested.

Adjusted covariates were pH, base deficit, Apgar score 5 min, placental abruption, where missing covariate values were imputed by the mean for each group (Table 4). The correlations of blood sLOX-1 with the severity groups were examined using Spearman’s rank correlation coefficient and a Wilcoxon rank sum test (Table 5). The distribution of sLOX-1 is shown as a boxplot in Figures S1–S3. Statistical analysis was performed using a t-test and a Mann–Whitney test, with significant differences indicated by *p* < 0.05.

## 3. Results

Of 193 participants, one case each of congenital duodenal atresia and partial deletion of the long arm of chromosome 20, both of which were diagnosed after hospitalization, was excluded, and the remaining 191 cases were included in the study. We collected 191 samples, including 88 normal samples (50 from umbilical cord (UC) arteries and 38 venous blood samples from the patients’ bodies (CTL)), and 103 HIE samples (34 mild, 55 moderate, and 14 severe according to Sarnat staging). All of HIE samples were arterial or venous blood. The healthy participants included 50 babies presenting with no problems at birth at an obstetrics clinic. The 38 CTL participants were patients admitted to the NICU without a diagnosis of HIE; 18 were diagnosed with transient tachypnea, five with pneumothorax, and three with respiratory failure. All blood samples were collected within 6 h after birth before any treatments. The participants with moderate and severe HIE underwent therapeutic hypothermia after blood sampling.

### 3.1. Maternal and Neonatal Clinical Features

Maternal and neonatal clinical features are presented in Table 1 and Table 2. There were no significant differences in maternal conditions between the UC and CTL groups. Moreover, age, gravida, and parity exhibited no significant differences between groups, while placental abruption, non-reassuring fetal status (NRFS), and emergency cesarean delivery demonstrated significant differences between the UC and CTL groups and the HIE group. These results indicate that fetal heart rate abnormality may represent a risk factor for HIE due to placental dysfunction and could lead to emergency cesarean section.

The clinical features of all neonatal participants at delivery or upon admission to the neonatal care unit (NICU) are shown in Table 2. There were no differences in birth weight and gestational age between all groups. The majority of participants in the HIE groups were admitted to private obstetrics clinics without neonatal care and transported to medical facilities with NICUs. It is reasonable that the APGAR score, intubation and/or resuscitation procedures, blood gas pH, and base deficit of the UC and CTL groups are significantly higher than those of the HIE groups. The results of the APGAR scores and blood gas tests, as well as resuscitation for 10 min or more, which are also used as diagnostic criteria for HIE, showed significant differences between the HIE group and healthy newborns, with the differences being more pronounced the more severe the condition. The blood examination indicated that creatine kinase (CK) and sLOX-1 levels were significantly different between all groups, suggesting that these two biomarkers may help to diagnose HIE. Interestingly, sLOX-1 values correlated with HIE severity (Figure S1), and platelet counts, CK, and lactate dehydrogenase (LDH) levels exhibited significant differences between mild HIE and moderate and severe HIE.

Table 3 outlines the clinical condition of participants during neonatal care hospitalization and at discharge. In the HIE group, the patients underwent some kind of intervention or treatment, such as therapeutic hypothermia, oxygen inhalation, or mechanical ventilation. The results indicate that almost all neonates with moderate and severe HIE underwent therapeutic hypothermia and ventilation, and show a high rate of death in those with severe HIE. Neurosensory impairments and other complications were observed as sequela of moderate and severe HIE, with the severity and frequency of these symptoms, as well as the duration of hospitalization, oxygen inhalation, ventilation, and feeding, increasing with rising HIE severity.

### 3.2. Statistical Analyses of sLOX-1 Values and Sarnat Staging

The distribution of sLOX-1 is shown in Figure S1 and Table 2. The mean sLOX-1 value in the UC group was 497 pg/mL (interquartile range (IQR): 337–693 pg/mL), 613 pg/mL (IQR: 474–925 pg/mL) in the CTL group, 1030 pg/mL (IQR: 658–1547 pg/mL) in the mild HIE group, 1026 pg/mL (IQR: 710–2051 pg/mL) in the moderate HIE group, and 1444 pg/mL in the severe HIE group (IQR: 766–3927 pg/mL). We detected four outliers in the moderate and severe HIE groups, so we logarithmically transformed the sLOX-1 values for the statistical analyses (Table 4 and Table 5).

There was no significant difference between the UC and CTL groups (*p* = 0.136, Figure S1 and Table 4). However, there were significant differences between the UC and HIE groups and between the CTL and HIE groups (*p* = 0.006, Figure S1, Table 4 and Table 5). Figure S2 showed the advantage of sLOX-1 measurement alongside the Sarnat score to facilitate the decision to administer therapeutic hypothermia, with therapeutic hypothermia generally employed for babies with moderate and severe HIE. However, the Area Under the Curve (AUC) by the Receiver Operating Characteristic curve (ROC) was 0.628.

In addition, we also confirmed significant differences in groups of patients who were clinically intact at the time of discharge and those with neurological and/or other sequelae or death at discharge (Figure S3).

## 4. Discussion

The present study demonstrated that blood sLOX-1 values within 6 h after birth were correlated with Sarnat staging. Additionally, there was a significant difference in sLOX-1 levels between the CTL and HIE groups, indicating that it is a useful biomarker for the diagnosis of HIE. We found that sLOX-1 levels parallel the severity of HIE and might be useful for discriminating babies with moderate and severe HIE from healthy babies and for deciding whether hypothermia therapy should be administered. The high levels of sLOX-1 may indicate moderate or severe brain damage. We also found that sLOX-1 levels decline rapidly from 24 h after birth in both infants with HIE and healthy infants. This is consistent with our previous pilot study and a previous study on acute coronary syndrome [7,14].

As a novel biomarker, early blood sLOX-1 levels, alongside the clinical severity of HIE and neurologic symptoms within approximate one-month old, may be useful for severity staging and as a short-term prognostic biomarker of HIE. We speculated that blood sLOX-1 levels would be high in infants with HIE, given the high sLOX-1 levels previously observed in a rat model of HIE (unpublished data). In fact, sLOX-1 levels in the first 6 h after birth tended to increase in a stepwise manner with increasing HIE severity, in accordance with a previous report linking sLOX-1 levels to the number of lesions in acute coronary syndrome [14]. Moreover, sLOX-1 levels differed between infants with intact survival and those who died or exhibited neurosensory impairment from the result of Figure S3. However, we cannot effectively set the cutoff value because of the AUC of 0.7428. In the future, an increase in the number of patients with severe HIE is expected to enable the determination of a meaningful cutoff value.

Previous reports on the long-term prognosis of HIE (at 18 months of age) showed neurosensory impairment in 22% of the mild HIE group, and the frequency of death or neurosensory impairment was reported to be 44% to 55% in the moderate and severe HIE groups treated with hypothermia [18,19]. Applying these to the present study, it is estimated that of the included patients, approximately 38 to 45 cases of neurosensory impairment or death will occur. Of the cases who are intact at the time of discharge, approximately 20 may be diagnosed with neurosensory disorders as they grow older. The results of this study suggest that it is difficult to determine whether sLOX-1 levels at birth are intact at the time of discharge, but a correlation between sLOX-1 levels at birth and long-term prognosis may be observed based on the results of developmental tests at 18 months and 3 years of age after discharge. When applied to the patients without therapeutic hypothermia, blood sLOX-1 levels may better predict death or neurosensory impairment.

The placenta strongly expresses LOX-1, which may explain the physiologically increased sLOX-1 levels seen in control group infants at birth [15]. All groups included cases of maternal hypertension due to pregnancy, but the proportion was small, and no significant differences were observed between the groups in Table 1. Therefore, in this study, it was predicted that maternal hypertension during pregnancy would have little effect on the comparison of sLOX-1 in umbilical cord blood or infant blood between each group. In addition, the number of placental abruption did not change significantly in the UC and CTL groups, but increased significantly in the HIE groups. All blood samples were derived directly from newborns and not from potentially contaminated placental blood. Therefore, there is little possibility that fetal stress from maternal distress induced LOX-1. However, there were no significant differences between each HIE group. Thus, we can speculate that there was no need to consider placenta factors. On the other hand, microglia markedly expressed LOX-1 in an HIE rat model and an in vitro HIE model [20]. The blood sLOX-1 value may reflect brain damage primarily from activated microglia as a result of hypoxic–ischemic insult.

Early severity staging of HIE is important for ensuring the initiation of therapeutic hypothermia within the first 6 h after birth [21,22,23,24]. MRI and amplitude-integrated electroencephalography (aEEG) may be useful as biomarkers of HIE. The NICHD MRI brain injury pattern of HIE is simple and closely correlated with the developmental outcome of HIE [17,19]. In our cohort, all infants with the most severe brain injury had a poor outcome. However, MRI may not be appropriate for severity staging in the acute phase of HIE. In a meta-analysis, it was reported that a persistently abnormal aEEG 48 h after birth or later was related to poor outcomes, but that aEEG 6 h after birth was not related to outcomes [24]. Our data suggest that sLOX-1 may be sensitive to these physiological biomarkers, given that levels were associated with both early severity of HIE and neurosensory impairments. HIE pathophysiology is thought to include ischemia, followed by reperfusion and induction of cytokines, the activation of which induces functional deficit and neuronal apoptosis [25,26,27]. LOX-1 can be induced by the same condition in early HIE. Microglia are related to neural inflammation in HIE, and their activation is induced by hypoxic–ischemic insult [18]. It may be speculated that LOX-1 is expressed in endothelial cells and microglia of the human brain during HIE. Thus, it is considered that hypoxic–ischemic insult induces LOX-1 up-regulation and activation in microglia. LOX-1 activation induces cytokines or other stress materials through self-positive feedback and enhances cytotoxic inflammation in neonatal brains.

We propose a potential HIE treatment using LOX-1 inhibitors and neutralizing antibodies. BI-0115, a selective small-molecule inhibitor of LOX-1, blocks cellular uptake of oxLDL [28], and rat ischemic stroke models were found to exhibit improved neurological function following combination therapy of rt-PA (recombinant tissue-type plasminogen activator) and BI-0115 and/or MMP-9 (matrix metalloproteinase 9) [29]. Another study using an HIE rat model showed pathological and biological improvement following antibody treatment [17]. Thus, we believe that early diagnosis of HIE and early treatment with LOX-1 inhibitor(s) or neutralizing antibodies could improve rates of intact survival.

The expression of LOX-1 has been found to be closely related to the pathology of brain injuries in rats and humans with HIE. The combination of these factors with sLOX-1 may serve as a powerful biomarker of HIE. As such, sLOX-1 levels may be useful for deciding whether to induce hypothermia and/or other treatments, as well as for evaluating the under- and overtreatment of patients, making prognoses, and planning medical care.

We did not find clearly stratified relationships between mild and moderate HIE or between moderate and severe HIE. Therefore, confirmation of sLOX-1 as a useful staging biomarker of HIE requires further studies with larger samples. In addition, further assessment of long-term outcomes in a study with multiple hospitals would support the usefulness of sLOX-1 as a diagnostic and prognostic biomarker of HIE. The results of this study suggest that early blood sLOX-1 measurement in combination with the Sarnat system could assist in determining HIE severity.

## 5. Limitations

In this study, we could only obtain a small number of patients with severe HIE, because its incidence is extremely rare in Japan. Therefore, the results might be insufficient to draw conclusions about severe HIE cases. In addition, while the timing of blood sampling was within 6 h after birth, it was difficult to determine the exact sampling times while performing procedures and administering treatment in the NICU. However, the results of this study would support HIE diagnosis and consideration of HIE severity.

The observation interval of this study was also limited to one to just a few months. The long-term neurological and developmental follow-up study is underway to confirm the diagnostic value of blood sLOX-1.

## Figures and Tables

**Table 1:** Maternal clinical features.

				HIE (Sarnat Staging)			Statistical Differences (Between Each Group) (*p*-Value)				
		Umbilical Cord (UC)	Control (CTL)	Mild	Moderate	Severe	All *	UC vs. CTL **	UC vs. HIE **	CTL vs. HIE **	Mild vs. Moderate + Severe **
	age (years, (mean ± SD))	34.3 ± 4.4	33.0 ± 3.9	34.4 ± 3.8	33.2 ± 5.1	34.0 ± 3.0	0.498	0.165	0.587	0.314	0.458
	gravida (median (IQR))	2 (1–2)	2 (1–3)	2 (1–3)	1 (1–2.25)	1 (1–2)	0.228	0.469	0.410	0.089	0.097
	parity (median (IQR))	1 (0–2)	0 (0–1)	0.5 (0–1)	0 (0–1)	0 (0–1)	0.553	0.359	0.133	0.667	0.330
	non-reassuring fetal status (N (%))	11 (23.4)	12 (31.6)	24 (72.7)	46 (88.5)	13 (92.6)	**<0.001**	0.399	**<0.001**	**<0.001**	**0.034**
	emergency cesarean delivery (N (%))	11 (22)	10 (26.3)	13 (39.4)	37 (67.3)	8 (61.5)	**<0.001**	0.688	**<0.001**	**0.001**	**0.011**
Complication of pregnancy (N (%))											
	total	5 (10.6)	6 (15.8)	11 (32.4)	17 (31.5)	4 (28.6)	0.172	0.351	**0.006**	0.065	0.880
	hypertension	2 (4.3)	5 (13.2)	4 (11.8)	7 (12.7)	1 (7.1)	0.707	0.139	0.124	0.505	0.607
	diabetes	3 (6.4)	2 (5.3)	1 (2.9)	2 (3.6)	2 (14.3)	0.485	0.601	0.484	0.608	0.464
	placental abruption	0 (0)	0 (0)	10 (29.4)	10 (18.2)	2 (14.3)	0.099	-	**0.001**	**0.002**	0.162
	pre-eclampsia	0 (0)	0 (0)	0 (0)	0 (0)	0 (0)	-	-	-	-	-
Intrapartum complications (N (%))											
	total	12 (25.5)	15 (40.5)	24 (72.7)	47 (88.7)	13 (92.9)	**<0.001**	0.144	**<0.001**	**<0.001**	**0.031**
	non-reassuring fetal status	11 (23.4)	12 (31.6)	24 (72.7)	46 (88.5)	13 (92.9)	**<0.001**	0.399	**<0.001**	**<0.001**	**0.034**
	cord prolapse	0 (0)	0 (0)	0 (0)	0 (0)	1 (7.7)	0.085	-	0.662	0.731	0.673
	coiling of cord	2 (4.0)	6 (16.7)	3 (9.4)	5 (9.4)	1 (7.7)	0.285	0.053	0.214	0.180	0.614
	maternal fever	0 (0)	1 (2.8)	0 (0)	4 (7.5)	1 (7.7)	0.189	0.419	0.123	0.487	0.132
	others	0 (0)	0 (0)	0 (0)	3 (5.7)	2 (15.4)	**0.012**	-	0.123	0.203	0.132

Abbreviations: HIE, hypoxic–ischemic encephalopathy; IQR, interquartile range; UC, umbilical cord; CTL, control; N, number. *; Analysis of Variance, **; *t*-test for continuous variables, Chi-square test or Fisher’s exact test for categorical variables. Bold, statistic significance.

**Table 2:** Clinical summary of participants at admission.

				HIE (Sarnat Staging)			Statistical Differences (Between Each Group) (*p*-Value)				
		Umbilical Cord (UC)	Control (CTL)	Mild	Moderate	Severe	All *	UC vs. CTL **	UC vs. HIE **	CTL vs. HIE **	Mild vs. Moderate + Severe **
	N	50	38	34	55	14					
	sex, male–female	21:29	26:12	21:13	27:28	3:11					
	birth weight (g, mean ± SD)	3089 ± 375	2922 ± 465	2833 ± 439	2946 ± 483	2958 ± 447	0.131	0.085	**0.032**	0.917	0.246
	gestational age (weeks, median (IQR))	39.2 (38.4–40.4)	39.0 (37.3–40.0)	39.2 (38.5–40.2)	39.4 (38.2–40.4)	38.5 (37.5–40.4)	0.076	-	0.295	0.044	0.897
	APGAR score, 1 min; 5 ≤ (N (%))	0 (0)	9 (23.7)	27 (79.4)	54 (98.2)	14 (100)	**<0.001**	-	**<0.001**	**<0.001**	**0.012**
	APGAR score, 5 min; 5 ≤ (N (%))	0 (0)	1 (2.6)	16 (47.1)	46 (83.6)	11 (78.6)	**<0.001**	-	**<0.001**	**<0.001**	**<0.001**
	APGAR score, 10 min; 5 ≤ (N (%))	0 (0)	0 (0)	5 (14.7)	18 (37.2)	9 (64.3)	**<0.001**	-	**<0.001**	**<0.001**	0.111
	intubation in delivery room (N (%))	0 (0)	0 (0)	10 (29.4)	31 (56.7)	10 (71.4)	**<0.001**	-	**<0.001**	**<0.001**	**0.004**
	resuscitation at 10 min (N (%))	0 (0)	2 (5.4)	24 (79.4)	48 (87.3)	12 (85.7)	**<0.001**	-	**<0.001**	**<0.001**	**0.031**
	blood gas value in first 60 min (pH, median (IQR))	7.34 (7.31–7.37)	7.24 (7.17–7.30)	7.18 (7.08–7.37)	7.06 (6.93–7.20)	6.87 (6.77–7.00)	**<0.001**	-	**<0.001**	**<0.001**	**<0.001**
	blood gas value in first 60 min (base deficit, median (IQR))	1.3 (0.1–3.1)	5 (3.1–7.0)	10 (5.5–13.5)	14.9 (11.9–19.8)	24.8 (16.1–27.7)	**<0.001**	-	**<0.001**	**<0.001**	**<0.001**
main blood examination at admission											
	RBCs (median (IQR)), ×1000	not examined	4375 (4162.5–5337.5)	4835 (4467.5–5132.5)	4555 (4097.5–4910)	4665 (4212.5–4750)	0.112	-	-	0.609	0.054
	WBCs (median (IQR))	not examined	20,505 (18,788–21,735)	19,285 (15,125–24,050)	19,700 (16,000–25,775)	20,300 (16,800–22,805)	0.926	-	-	0.755	0.562
	PLTs (median (IQR)), ×1000	not examined	270 (229.5–297)	286 (222–307.25)	226 (189.5–276)	213.5 (135.75–290.75)	0.098	-	-	0.501	**0.022**
	CK (median (IQR))	not examined	582 (406–624)	1284 (704–1936)	1141 (581–1758)	1276 (504–3294)	**0.029**	-	-	0.191	**0.029**
	LDH (median (IQR))	not examined	545 (520–578)	623 (526–779)	700 (608–1170)	823 (686–1162)	0.054	-	-	0.159	**0.037**
	CRP (median (IQR))	not examined	0.04 (0.01–0.09)	0.02 (0.01–0.07)	0.03 (0.01–0.10)	0.04 (0.02–0.30)	0.793	-	-	0.445	0.834
	sLOX-1 (pg/mL, (median (IQR))	497 (337–693)	613 (474–925)	1030 (658–1547)	1026 (710–2051)	1444 (766–3927)	**<0.001**	0.054	**<0.001**	**<0.001**	0.075

Abbreviations: HIE, hypoxic–ischemic encephalopathy; IQR, interquartile range; RBCs, red blood cells; WBCs, white blood cells; PLTs, platelets; CK, creatine kinase; LDH, lactate dehydrogenase; CRP, C-reactive protein; sLOX-1, soluble form of lectin-like oxidized LDL receptor-1. *; Analysis of Variance, **; *t*-test for continuous variables, Chi-square test or Fisher’s exact test for categorical variables. Bold, statistic significance.

**Table 3:** In-hospital and short-term outcomes (at approximately 1 month of age).

			HIE (Sarnat Staging)			Statistical Differences (Between Each Group) (*p*-Value)				
	Umbilical Cord (UC)	Control (CTL)	Mild	Moderate	Severe	All *	UC vs. CTL **	UC vs. HIE **	CTL vs. HIE **	Mild vs. Moderate + Severe **
death or neurosensory impairments (N (%))	0 (0)	0 (0)	1 (3.4)	5 (10.0)	10 (76.9)	**<0.001**	-	0.073	0.279	**0.017**
hypothermia (N (%))	0 (0)	0 (0)	11 (32.4)	55 (100)	13 (92.9)	**<0.001**	-	**<0.001**	**<0.001**	**<0.001**
oxygen inhalation (N (%))	1 (8.3)	32 (84.2)	27 (84.4)	53 (98.1)	13 (100)	**0.008**	-	**<0.001**	0.076	**0.013**
mechanical ventilation (N (%))	0 (0)	3 (7.9)	19 (55.9)	54 (98.2)	14 (100)	**<0.001**	-	**<0.001**	**<0.001**	**<0.001**
death (N (%))	0 (0)	0 (0)	0 (0)	0 (0)	5 (35.7)	**<0.001**	-	0.132	0.200	0.125
complications (N (%))										
total	0 (0)	0 (0)	3 (9.1)	15 (28.3)	8 (61.5)	**<0.001**	-	**0.033**	**<0.001**	**0.006**
persistent pulmonary hypertension	0 (0)	0 (0)	3 (9.1)	5 (9.49	1 (7.7)	0.143	-	0.343	0.055	0.632
disseminated intravascular coagulation	0 (0)	0 (0)	1 (3.0)	7 (13.2)	5 (38.5)	**<0.001**	-	0.206	**0.012**	**0.030**
sepsis	0 (0)	0 (0)	0 (0)	0 (0)	0 (0)	-	-	-	-	-
multiorgan failure	0 (0)	0 (0)	0 (0)	0 (0)	3 (23.1)	**0.002**	-	0.707	0.374	0.292
neurosensory impairments (N (%))										
total	0 (0)	0 (0)	1 (3.4)	5 (10.0)	8 (72.7)	**<0.001**	-	0.099	0.323	**0.024**
feeding problems	0 (0)	0 (0)	1 (2.9)	3 (5.6)	6 (46.2)	**<0.001**	-	0.236	**0.036**	0.089
seizure requiring anticonvulsants	0 (0)	0 (0)	0 (0)	2 (3.7)	2 (18.2)	**0.010**	-	0.561	0.265	0.187
hearing problems	0 (0)	0 (0)	0 (0)	0 (0)	5 (55.6)	**<0.001**	-	-	0.676	0.128
hypotonia	0 (0)	0 (0)	0 (0)	0 (0)	1 (10.0)	0.069	-	0.866	0.719	0.660
length of hospitalization (days, median (IQR))	6 (6–8)	8 (6–12)	18 (13–21)	24.5 (17.3–33.8)	39 (25.3–75.3)	**<0.001**	0.582	**<0.001**	**<0.001**	**<0.001**
length of oxygen inhalation (days, median (IQR))	0 (0–0)	2 (1–4)	4 (1–7.3)	6 (5–9)	13.5 (9.5–23)	**<0.001**	0.610	**0.008**	**0.001**	**<0.001**
length of mechanical ventilation (days, median (IQR))	0 (0–0)	0 (0–0)	1 (0–4.8)	5 (5–6)	7.5 (5–17.8)	**<0.001**	0.953	**0.019**	**<0.001**	**<0.001**
full feeding (days, median (IQR))	4 (4–4)	4 (3–5)	6 (5–8)	8 (7–9)	11 (9–23)	**<0.001**	0.991	**0.003**	**<0.001**	**<0.001**

Abbreviations: HIE, hypoxic–ischemic encephalopathy; IQR, interquartile range. *; Analysis of Variance, **; *t*-test for continuous variables, Chi-square test or Analysis of Variance for categorical variables. Bold, statistic significance.

**Table 4:** Adjusted analyses of blood sLOX-1 values and HIE severity.

	Contrast Estimate (95% Confidence Interval)	Two-Sided *p*-Value
Global	-	<0.001
UC vs. CTL	0.29 (−0.09, 0.68)	0.136
UC vs. HIE (mild + moderate + severe) *	1.05 (0.42, 1.69)	0.001
CTL vs. HIE (mild + moderate + severe) *	0.76 (0.22, 1.30)	0.006
UC + HIE (mild) vs. HIE (moderate + severe) **	0.77 (0.28, 1.26)	0.002
CTL + HIE (mild) vs. HIE (moderate + severe) **	0.62 (0.17, 1.07)	0.007
HIE (mild) vs. HIE (moderate + severe) ***	0.36 (−0.05, 0.78)	0.088
Linear trend among UCB, HIE (mild), HIE (moderate), and HIE (severe) ****	4.39 (1.82, 6.95)	0.001
Linear trend among CTL, HIE (mild), HIE (moderate), and HIE (severe) ****	3.50 (1.18, 5.83)	0.003

Severity (normal (CTL), mild, moderate, and severe) is based on modified Sarnat classification. Adjusted variables: blood gas value (pH), blood gas value (base deficit), APGAR score (5 min), placental abruption. Abbreviations: HIE, hypoxic–ischemic encephalopathy; IQR, interquartile range; UC, control of umbilical cord artery; CTL, control. *: Contrast coefficients (Normal (CTL), Mild, Moderate, Severe) = (−1, 1/3, 1/3, 1/3). **: Contrast coefficients (Normal (CTL), Mild, Moderate, Severe) = (−0.5, −0.5, 0.5, 0.5). ***: Contrast coefficients (Normal (CTL), Mild, Moderate, Severe) = (0, −1, 0.5, 0.5). ****: Contrast coefficients (Normal (CTL), Mild, Moderate, Severe) = (−3, −1, 1, 3).

**Table 5:** Correlation of blood sLOX-1 values with severity groups.

Spearman’s Rank Correlation Coefficient		Z Value or Correlation Coefficient (95% CI)	*p*-Value
test between UC and each group		0.43 (0.30, 0.55)	<0.001
	UC vs. CTL *	2.39	0.017
	UC vs. Mild *	4.41	<0.001
	UC vs. Moderate *	4.72	<0.001
	UC vs. Severe *	2.68	0.007
test between UC + CTL and each group		0.41 (0.27, 0.53)	<0.001
	UC + CTL vs. Mild *	4.17	<0.001
	UC + CTL vs. Moderate *	4.57	<0.001
	UC + CTL vs. Severe *	2.58	0.01
test between CTL and each group		0.22 (0.04, 0.39)	0.019
	CTL vs. Mild *	−2.22	0.026
	CTL vs. Moderate *	−2.34	0.019
	CTL vs. Severe *	1.9	0.057

Abbreviations: UC; umbilical cord, CTL; control. * Wilcoxon rank sum test. Severity (normal (CTL), mild, moderate, and severe) is based on modified Sarnat classification.

## Data Availability

The original contributions presented in this study are included in the article/Supplementary Materials. Further inquiries can be directed to the corresponding author.

## Supplementary Materials

The following supporting information can be downloaded at https://www.mdpi.com/article/10.3390/medsci14030391/s1: Figure S1: sLOX-1 distribution of participants within 6 h after birth; Figure S2: sLOX-1 levels for therapeutic hypothermia; Figure S3: Correlation of short-term outcomes and sLOX-1 values.

## Abbreviations

The following abbreviations are used in this manuscript:

sLOX-1	soluble form of lectin-like oxidized low-density lipoprotein receptor-1
HIE	neonatal hypoxic–ischemic encephalopathy
CTL	control
oxLDL	low-density lipoprotein
ABR	automated auditory brainstem response
UC	umbilical cord
NICU	Neonatal Intensive Care Unit
IQR	interquartile range
aEEG	amplitude-integrated electroencephalography
