# Supplementary material for: Blood sLOX-1 Is Beneficial for Determining Severity of Neonatal Hypoxic–Ischemic Encephalopathy: A Nationwide Prospective Cohort Study
Source: Med Sci (Basel). 2026 Jul 14;14(3):391. doi: 10.3390/medsci14030391 (PMC13413686; doi:10.3390/medsci14030391)
Supplement: Supplementary file 1 [file medsci-14-00391-s001.zip › medsci-4354177-supplementary.pdf]

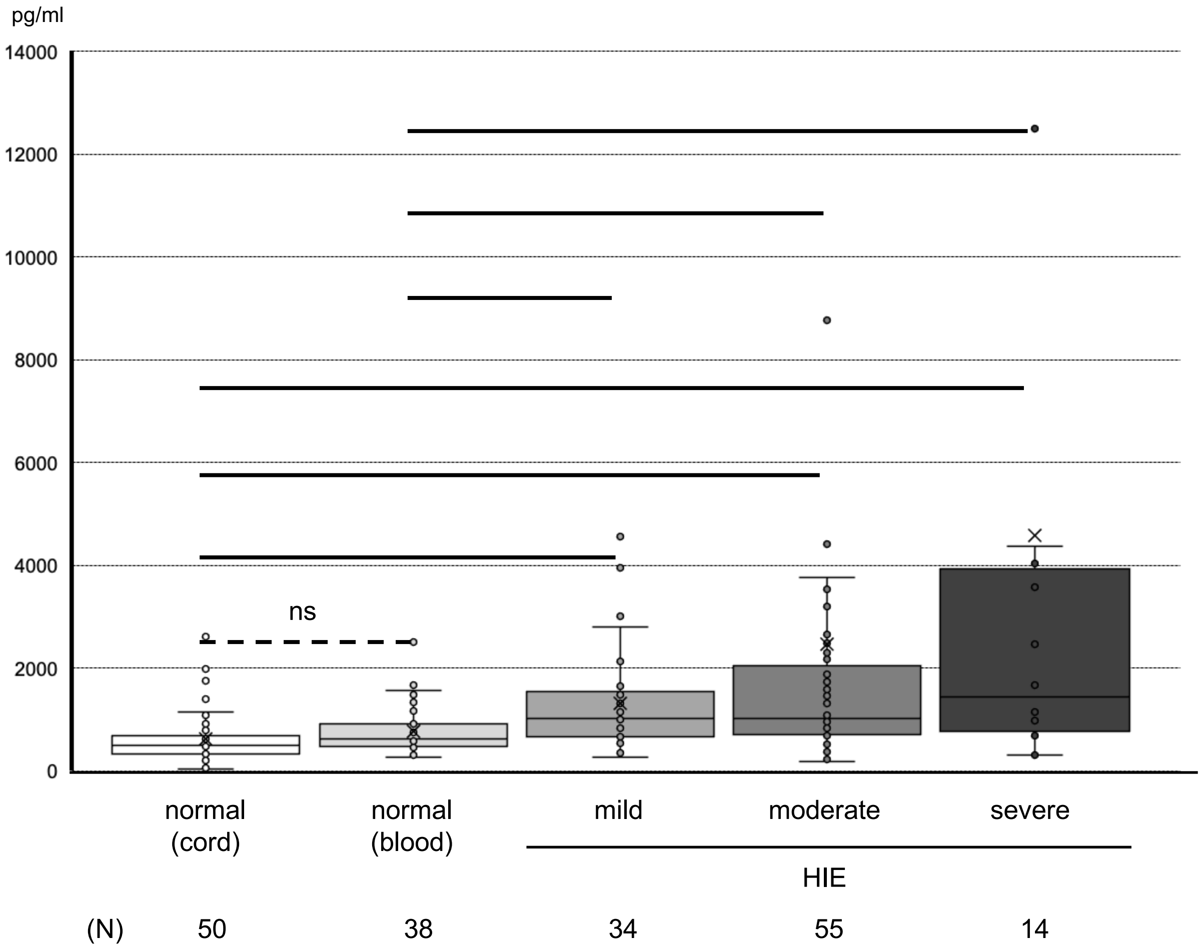


Figure S1. sLOX-1 distribution of participants within 6 h after birth .

Plasma sLOX-1 values of 191 participants significantly increases with severity. However, there is no change of sLOX-1 values of normal umbilical cord and venous bloods.

t-test for continuous variables, —— : *P*<0.05, – – : ns (not signiicant).

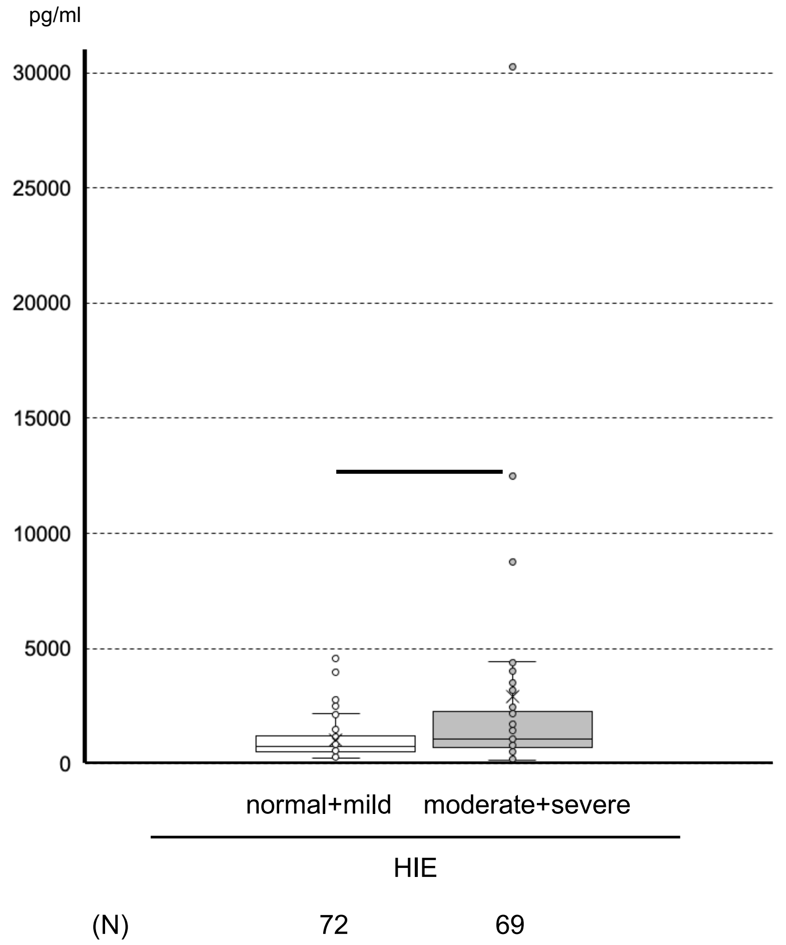


Figure S2. sLOX-1 levels for therapeutic hypothermia .

There is significant difference of sLOX-1 values between a normal and mild HIE group and a moderate and severe HIE group.

t-test for continuous variables, —— : $P<0.05$.

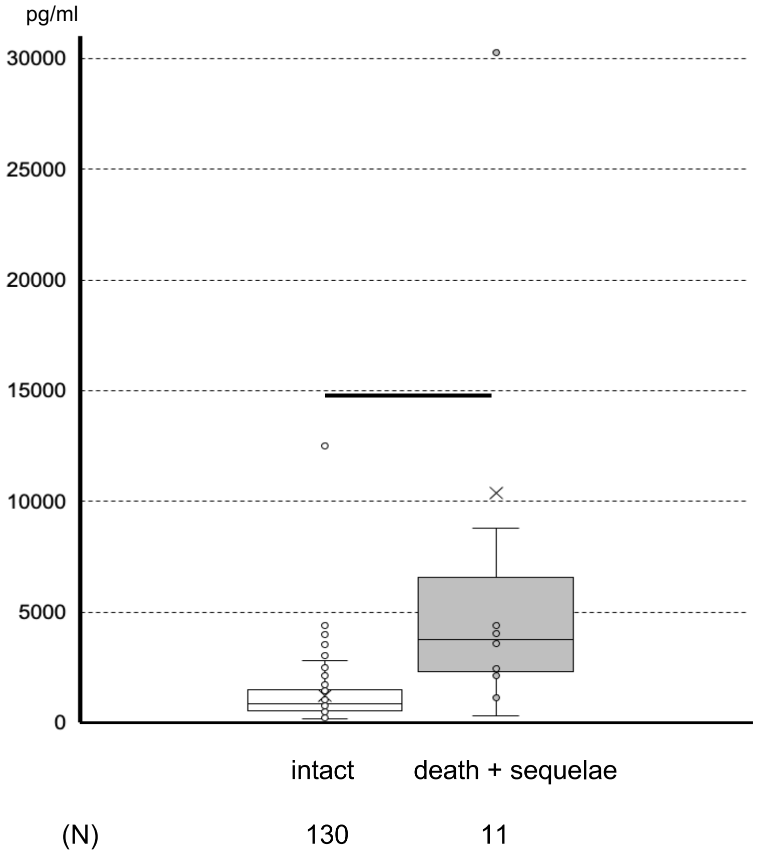


Figure S3. Correlation of short-term outcomes and sLOX-1 values .
Approximately one month after birth, we confirmed 11 death or neurological sequelae and their sLOX-1 values were significantly higher than those of 130 intact participants.

t-test for continuous variables, —— : $P<0.05$.
